# Trimethyl Chitosan Nanoparticles Encapsulated Protective Antigen Protects the Mice Against Anthrax

**DOI:** 10.3389/fimmu.2018.00562

**Published:** 2018-03-20

**Authors:** Anshu Malik, Manish Gupta, Rajesh Mani, Himanshu Gogoi, Rakesh Bhatnagar

**Affiliations:** ^1^Molecular Biology and Genetic Engineering Laboratory, School of Biotechnology, Jawaharlal Nehru University, New Delhi, India

**Keywords:** anthrax, vaccine, protective antigen, trimethylchitosan nanoparticles, CpG, PolyI:C

## Abstract

Anthrax is an era old deadly disease against which there are only two currently available licensed vaccines named anthrax vaccine adsorbed and precipitated (AVP). Though they can provide a protective immunity, their multiple side-effects owing to their ill-defined composition and presence of toxic proteins (LF and EF) of *Bacillus anthracis*, the causative organism of anthrax, in the vaccine formulation makes their widespread use objectionable. Hence, an anthrax vaccine that contains well-defined and controlled components would be highly desirable. In this context, we have evaluated the potential of various vaccine formulations comprising of protective antigen (PA) encapsulated trimethyl-chitosan nanoparticles (TMC-PA) in conjunction with either CpG-C ODN 2395 (CpG) or Poly I:C. Each formulation was administered *via* three different routes, viz., subcutaneous (SC), intramuscular (IM), and intraperitoneal in female BALB/c mice. Irrespective of the route of immunization, CpG or Poly I:C adjuvanted TMC-PA nanoparticles induced a significantly higher humoral response (total serum IgG and its isotypes viz., IgG1, IgG2a, and IgG2b), compared to their CpG or Poly I:C PA counterparts. This clearly demonstrates the synergistic behavior of CpG and Poly I:C with TMC nanoparticles. The adjuvant potential of TMC nanoparticles could be observed in all the three routes as the TMC-PA nanoparticles by themselves induced IgG titers (1–1.5 × 10^5^) significantly higher than both CpG PA and Poly I:C PA groups (2–8 × 10^4^). The effect of formulations on T-helper (T_h_) cell development was assessed by quantifying the Th1-dependant (TNF-α, IFN-γ, and IL-2), Th2-dependant (IL-4, IL-6, and IL-10), and Th17-type (IL-17A) cytokines. Adjuvanation with CpG and Poly I:C, the TMC-PA nanoparticles triggered a Th1 skewed immune response, as suggested by an increase in the levels of total IgG2a along with IFN-γ cytokine production. Interestingly, the TMC-PA group showed a Th2-biased immune response. Upon challenge with the *B. anthracis* Ames strain, CpG and Poly I:C adjuvanted TMC-PA nanoparticles immunized *via* the SC and IM routes showed the highest protective efficacy of ~83%. Altogether, the results suggest that CpG or Poly I:C adjuvanted, PA-loaded TMC nanoparticles could be used as an effective, non-toxic, second generation subunit-vaccine candidate against anthrax.

## Introduction

*Bacillus anthracis*, a Gram-positive microorganism, is the cause of an acute disease Anthrax. It can form spores, which could remain dormant for years and accidental exposure by inhalation may result in death (1). Although inhalational anthrax has been reported to have a high fatality rate near to 100%, few deaths have also been documented due to cutaneous and gastrointestinal anthrax (2). It is also recognized for many bioterror attacks in history, including the famous 2001 bioterror attack of anthrax spores in the US (3). A prophylactic approach would be the best way to cease the disease and protect people against any bioterror attack. The currently existing vaccines BioThrax (also known as anthrax vaccine adsorbed) and anthrax vaccine precipitated (AVP) are the only licensed vaccines present globally (4). The major caveats of these vaccines are the presence of toxic proteins [lethal factor (LF) and edema factor (EF)] and the undefined composition of anthrax proteins (5). At present, only the high-risk population such as defense personnel are vaccinated with BioThrax. However, there have been several reports of reactogenicity in such individuals (6). Another drawback associated with this vaccine is the cumbersome dosing schedule, which comprises of multiple initial doses and subsequent annual boosters for sustained immunity (7).

In recent times, the focus has shifted to second generation subunit vaccines where the composition is pharmaceutically defined, and highly immunogenic proteins are taken into account making it safer for the mass use. In case of anthrax, *B. anthracis*, the causative organism of anthrax, secrete two toxins comprised of three proteins: the protective antigen (PA), the LF, and the EF. The edema toxin (PA + EF) causes edema and lethal toxin (PA + LF) causes death in animals (8). PA has always been the choice of antigen as it binds to the host receptor and binds to LF and EF to make lethal toxin and edema toxin, which eventually cause the toxicity in the host (9). Hence, the antibodies against PA have been shown to protect against aerosolized *B. anthracis* Ames spore challenge (10).

A soluble antigen when injected alone has less residence time and often lack essential danger signals necessary to provoke dendritic cells and consequently, T-cells. To surpass these limitations, antigen encapsulation into particulate system helps by increasing the retention time of the antigen in the host system and creating a depot, which releases the antigen into the system for a longer duration in a controlled manner (11, 12). The susceptibility of particles to be phagocytized by the macrophages is 1,000–10,000 times more efficient, and it also allows multimeric antigen presentation and delivery to antigen-presenting cells (APCs), in comparison to the soluble antigens (13).

The intervention of natural, biodegradable polymers-based encapsulation carriers makes them reasonably safe to employ for vaccine delivery (14, 15). Poly (lactic-co-glycolic acid) and chitosan are the most studied among them. Chitosan is derived from chitin, which is a ubiquitous natural polymer and is soluble in water under acidic conditions. Chitosan particles are usually positively charged and irregular in shape. However, they are susceptible to dissociation with ionic fluctuations and unstable at physiological pH. Several chemically modified (at functional groups -OH and -NH2) derivatives of chitosan have been studied. For instance, trimethyl-chitosan (TMC) is preferred over chitosan due to its stability at several pH ranges and its positive surface charge and has been utilized for vaccine delivery against influenza and Hepatitis B (14, 16–21).

Trimethyl-chitosan as well as chitosan have also been claimed to possess mucoadhesive and intrinsic adjuvant properties, and evidently, its nanoparticles can stimulate *in vitro* T-cell maturation and proliferation (22). Furthermore, it also possesses the ability to open tight junctions and cross epithelial barriers by redistributing the cytoskeletal F-actin and ZO-1 tight junction protein (23–26). The precursor of TMC and chitosan, chitin, has also been demonstrated to be a size-dependent pathogen-associated molecular pattern (PAMP) and shown to interact and activate the macrophages through different TLRs and dectin-1 (27, 28).

In addition to TMC nanoparticles, we sought to investigate further whether the immune response of nanoparticles could be synergistically improved upon addition of an adjuvant. Alum or aluminum salts-based adjuvants are currently licensed by FDA for human use and are most commonly employed in combination with several antigens. Despite this, they too suffer from various shortcomings such as variable antigen adsorption, induction of weak T-cell-mediated immune response, poor maturation of APCs, and intermittent occurrence of hypersensitivity reactions (29–31). Hence, PAMP-based adjuvants CpG-C ODN 2395 and Poly I:C were used in the study, which has been documented to provide protective immunity against anthrax infection (32, 33). CpG interacts with the TLR9 receptor and initiates a cascade of immuno-stimulatory signaling events culminating in secreting various cytokines and chemokines. This, in turn, causes maturation, differentiation, and proliferation of several immune cell types and eventually perpetuates a pro-inflammatory and TH1-biased immune response (34, 35). Similarly, the interaction of Poly I:C with TLR-3 induces dendritic cells maturation and is also reported to promote the *in vitro* survival of activated CD4+ cells and *in vivo* survival of antigen-activated CD8+ cells (36–39). Cytokines also perform a crucial part in the generation of humoral and cell-mediated immunity, and hence were examined in this study post immunization. The inflammatory Th1 cytokines, comprising of IFN-γ, TNF, and IL-2, stimulate predominantly cell-mediated immunity and T-cell proliferation, along with T-helper cell differentiation (40, 41). Immune modulating Th2 cytokines, such as IL-4, IL-6, and IL-10, support humoral immunity and adaptive immunity, mediate allergic diseases, and regulate chronic infections (42, 43).

The following parameters are known to affect the immune response elicited by the particulate-antigen delivery system: (i) administration route, (ii) booster administration, and the interval between boosters (iii) delivery system and amount of antigen, (iv) inclusion of immune modulating molecules like toll-like receptors (TLRs) ligands. Hence, in this study, we evaluated the vaccine potential of various TMC-PA nano-formulations with adjuvants through subcutaneous (SC), intramuscular (IM), and intraperitoneal (IP) delivery platforms. The protective efficacy of the various TMC-PA vaccine formulations was also assessed.

## Materials and Methods

### Materials

Chitosan (75–85% deacetylated, mol wt 50–190 kDa), Sodium tripolyphosphate (TPP), Poly I:C and Tween 80 were purchased from Sigma (Sigma, India). CpG-C ODN 2395 was purchased from Hycult Biotech Pvt. Ltd. All other materials used were of the analytical or pharmaceutical grade.

### Preparation of TMC Form Chitosan

Trimethyl-chitosan was derived from chitosan as described previously by Muzarelli and Tanfani and Verheul et al. (17, 44), with some modifications. To begin with, for formic acid–formaldehyde methylation (Eschweiler-Clarke), 3 g of chitosan was dissolved in 10 ml of formic acid, 10 ml of formaldehyde, and 60 ml distilled water added one by one in a round bottom flask. Under reflux condensing conditions, it was heated in an oil bath at 70°C for 118 h, under constant magnetic stirring. The resultant viscous liquid was evaporated and treated with 1N NaOH to increase its pH to 12. The so-formed dimethyl-chitosan (DMC) was washed with distilled water and then dissolved in 25 ml of 1-methyl-2-pyrrolidinone in the presence of 0.5 ml of iodomethane. It was heated up to 40°C on an oil bath under constant magnetic stirring for overnight. The solution was then dissolved in ethanol/diethyl ether mixture (50:50) resulting in the precipitation of TMC. The precipitated TMC was separated by centrifugation and washed thrice with diethyl ether. After complete evaporation of diethyl-ether, TMC was dissolved in 50 ml of 10% NaCl and dialyzed against deionized water for 3 days. After dialysis, TMC was lyophilized to obtain a dried powder form. ^1^H-nuclear magnetic resonance (^1^H-NMR) spectroscopy was done to assess the presence of trimethyl groups in chitosan. The degree of quaternization (DQ) of the end product was estimated by:

(1) $$\text{DQ}\left(\text{\%}\right)=\frac{\left[{\left({\text{CH}}_{3}\right)}_{3}\right]}{\left[\text{H}\right]}\times 1/9\times 100$$

### Preparation of PA-loaded TMC Nanoparticles (TMC-PA)

The nanoparticles were prepared by using the ionic gelation method as described previously (22, 45). A schematic representation of the preparation process is illustrated by Figure S1 in Supplementary Material. Briefly, 10 mg of TMC was dissolved in 5 ml 10 mM HEPES (pH 7.4), and PA was also prepared in the same buffer (8 mg/ml). The protein was dissolved in the TMC solution achieving a final concentration of 0.1 mg/ml under constant stirring. While continuous stirring (350 rpm), 1 ml of the crosslinking agent, TPP solution (1.7 mg/ml) was added to the TMC-PA solution drop by drop to induce ionic complexation. Weight ratio TMC:PA:TPP of 10:0.5:1.7 was taken to get the desired size of particles. An opalescent dispersion forms after TPP addition indicating the formation of nanoparticles. The nanoparticles were collected by centrifugation at 12,000 rpm for 15 min on a 10 µl glycerol bed. The particles were stored at −20°C until further use. The supernatant obtained was used for Micro BCA protein estimation. The protein encapsulation efficiency of the particles was further calculated by using Eq. 2:

(2) $$\text{Encapsulation efficiency}\left(\text{\%}\right)=\frac{\begin{array}{l} \text{weight of encapsulated protein} \end{array}}{\begin{array}{l} \text{weight of total protein used for}\\ \text{encapsulation} \end{array}}\;\times 100$$

For size analysis, a separate batch of nanoparticles was prepared with a minor modification. 0.05% of Tween 80 was added after dissolving TMC in the HEPES buffer.

### Physical Characterization and *In Vitro* Release Profile of TMC-PA Nanoparticles

The particles were dissolved in HEPES buffer pH 7.4 to measure the size, polydispersity index and zeta potential of the particles with the help of a Nanosizer ZS apparatus (Malvern Instruments, Malvern, UK). For scanning electron microscopic (SEM) imaging, particles were coated on a carbon tape on an aluminum stub and then coated with gold particles at 2 kV for 200 s under inert Argon condition. SEM images were captured using an electron microscope [Zeiss EVO40 (Carl Zeiss, Thornwood, NY, USA)]. For transmission electron microscopy [JEM 2100 F (Jeol Ltd., Tokyo, Japan), Electron Microscopy Sciences, Hatfield, PA] the nanoparticles dissolved in HEPES buffer were dropped (20 µl) on a copper mesh grid (46). After evaporating the buffer, the grid was inserted in the instrument and imaging was done in a high vacuum, at 200 kV and direct magnification of 3,000X.

For the *in vitro* protein release profile of the nanoparticles, a definite amount of particles were dissolved in 1× PBS and were stirred at 100 rpm at 37°C in several aliquots. Each aliquot was taken out from stirring at a specific time point and was centrifuged to spin down the nanoparticles, and the supernatant was taken to estimate the protein released by the nanoparticles in that specific time interval. Consequently, at different time points (2, 4, 6, 8, 10, 12, 24, 48, and 96 h), the release of protein was measured to assess the release of nanoparticles. The protein release was monitored for 96 h.

### *In Vivo* Immunological Study

Mice were obtained from National Centre for Laboratory Animal Sciences, NIN, Hyderabad, India, and were maintained in animal holding room of BSL3 laboratory. All the animal experiments including challenge studies were performed in compliance with Institutional Animal Ethics Committee (Jawaharlal Nehru University) and Council for the Purpose of Control and Supervision of Experiments on Animals (CPCSEA, Ministry of Social Justice and Empowerment, Government of India). Female Balb/c mice (6–8 weeks of age) were immunized with the placebo nanoparticles (Blank NP), TMC-PA nanoparticles (TMC-PA), TMC-PA nanoparticles in combination with CpG (CpG TMC-PA), and Poly I:C (Poly I:C TMC-PA) (*n* = 21 per group). All these formulations were administered in mice *via* SC, IM and IP routes. The amount of PA administered with every formulation was 20 μg/mice. CpG (20 μg/mice) as well as Poly I:C (10 μg/mice) were mixed with the nanoparticles right before the administration into mice. After an initial prime dose, two successive booster doses were given at an interval of 7 and 15 days, respectively (as depicted in Figure S2 in Supplementary Material). Sera were collected on 28th and 42nd day and stored at −80°C until further use. The sera samples were then used for the estimation of total IgG, IgG1, and IgG2a endpoint antibody titer against PA (anti-PA) by ELISA. Administration routes used in the study and the corresponding volume of dose injected into each animal are detailed in Table S1 in Supplementary Material.

### Cytokine Analysis

After 42 days of primary immunization, three mice from each group were sacrificed, and their spleens were crushed to make a single cell suspension. 10^6^ cells were seeded in each well of a 96-well round bottom (Nunc) plate. The cells were stimulated with PA (10 µg/ml) to induce the production of cytokines in the supernatant. Unstimulated and Con A (5 μg/well) treated cells were taken as negative and positive control, respectively. After 48 h of stimulation, the supernatant was collected from each well and was used for cytokine measurement using CBA (Cytometric Bead Array) Mouse Th1/Th2/Th17 Cytokine Kit (BD Biosciences), according to manufacturer instructions. The data were acquired on a BD LSR™ II flow cytometer (Becton Dickinson) and analyzed using the FCAP Array software V3.0 (Becton 519 Dickinson).

### Survival Assay/Protective Efficacy Studies

At 43rd day, remaining mice in each group were challenged with 0.5 × 10^3^ spores of *B. anthracis* Ames strain. As a control, 20 µg of PA adsorbed on alum was subcutaneously injected into a separate mice group (*n* = 12) and then challenged simultaneously. Mice were monitored for 15 days for death events in each group. Survival curve was plotted to compare the efficiency of protection in vaccinated mice groups over control blank nanoparticle immunized mice group.

### Statistical Analysis

The results are reported as mean ± SE post data preparation and statistical analysis using GraphPad Prism v6.05 software. The statistical significance of antibody titer and cytokine level data was calculated by using one-way ANOVA, followed by Tukey’s multiple comparisons test. The survival curve for anthrax spore challenge experiment was evaluated using Kaplan–Meier survival estimates (GraphPad Prism, La Jolla, CA, USA). Statistically significant differences between the groups are highlighted by following denotations: * for *P*-value <0.05, ** for *P*-values <0.01, *** for *P*-values <0.001, and **** for *P*-values <0.0001.

## Results

### NMR Characterization of the Trimethyl Chitosan

First, a dimethyl derivative of chitosan (DMC) was obtained with formic acid and formaldehyde treatment. Followed by derivation of TMC from DMC, ^1^H-NMR spectrum was analyzed for the presence of trimethyl group in chitosan. The peak present at 3.33 ppm represents the trimethyl group incorporated into the polymer at the -NH2 group of chitosan, imparting a positive charge to the polymer (Figure S3 in Supplementary Material). The final product was completely soluble in water, and the NMR was carried out by dissolving the final product in D_2_O. The DQ calculated by the Eq. 1 above was 80.4 ± 5.2%. Negligible O-methylation was observed in the product as seen in the NMR profile.

### PA Encapsulated Nanoparticles Preparation

**Figure 1:**
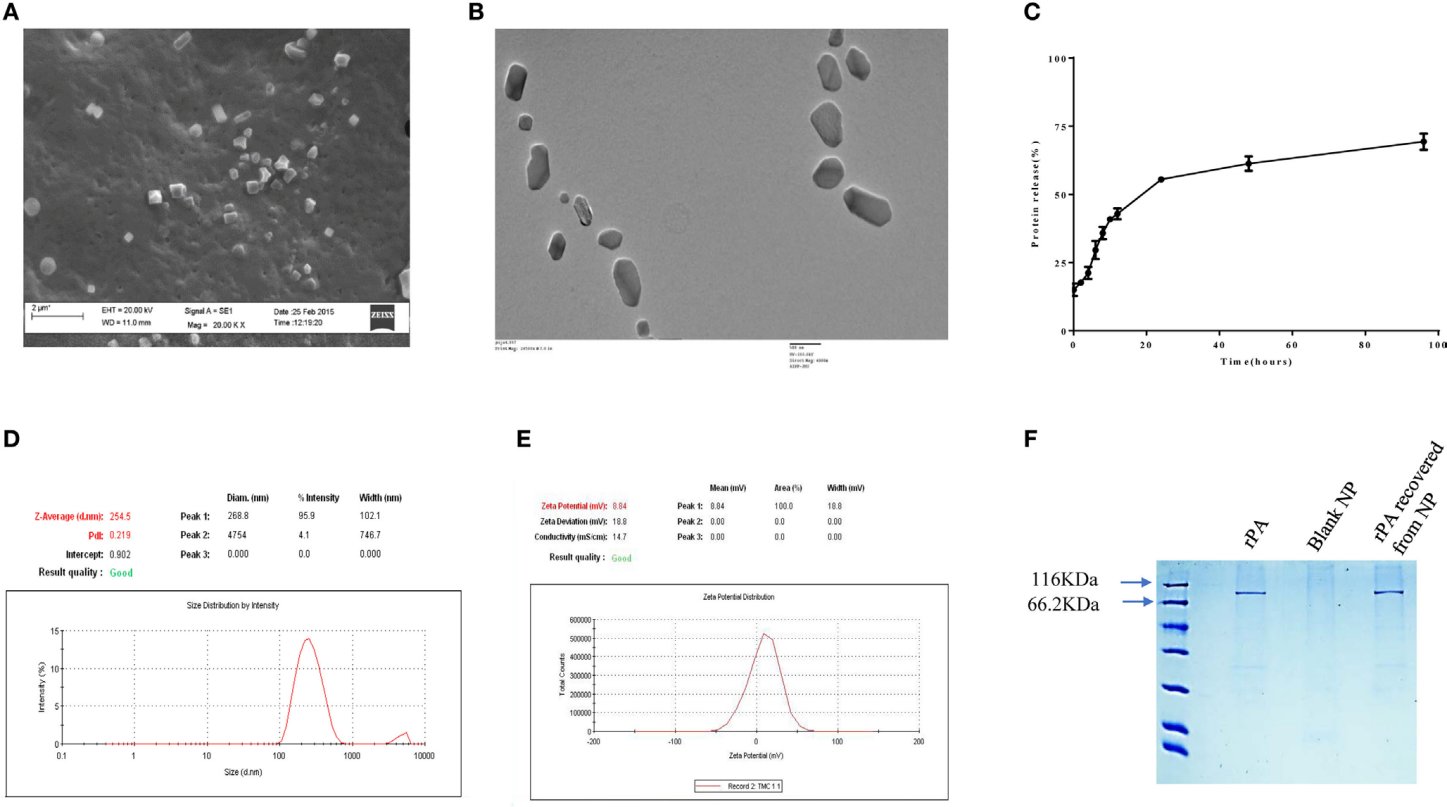
Physical characterization of nanoparticles. **(A)** scanning electron microscopic and **(B)** TEM micrographs, **(C)** *in vitro* release profile of protective antigen (PA) protein from Trimethyl-chitosan (TMC)-PA nanoparticles after suspension and incubation at 37°C in PBS (50 mg/ml, pH7.4) for indicated time periods and the released protein estimation by micro-BCA assay. **(D)** Particle size distribution (DLS) and **(E)** zeta potential of TMC nanoparticles encapsulating PA. **(F)** SDS PAGE analysis of the purified recombinant PA and the PA recovered from TMC-PA nanoparticles.

TMC-PA nanoparticles were prepared by ionic gelation method with the help of TPP anion. The particles were in the nanometer range with an average particle size of 254 ± 24 nm as depicted in Figure 1D. The particles shape and morphology was observed by SEM and TEM as shown in Figures 1A,B, and the nanoparticles were found to be smooth in texture but irregular in shape. The encapsulation efficiency of the particles was found to be 78.33 ± 4.75%. The polydispersity index of the particles was found to be 0.2 indicating fine homogeneity of the particles. Figure 1E reveals the zeta potential of the protein-loaded nanoparticles was found to be 8.84 ± 0.25 mV, which confirmed that the particles carried a positive charge.

### *In Vitro* Antigen Release

The antigen release profile of the TMC-PA nanoparticles was taken into account to estimate the amount of protein released from the particles over a specified period. The release of protein was studied until 96 h, illustrated in Figure 1C. Around 30% of the protein was released in the first 6 h only, and approximately 55% of the protein was released within first 24 h. Afterward, protein release was quite slow, and in next 24 h, only 7% protein was released. For the next 24 h also, only 8% release was observed. Therefore, in the later period, the release was slow and consistent inferring that remaining 30% of the protein would also be released at the same pace at these conditions. This suggests that at physiological pH, PA would be completely released within a week from the particles. Since 50% of the protein was burst released in the first 24 h; therefore, for immunization studies, 20 µg of protein encapsulating nanoparticles were used per mice. The encapsulated protein was analyzed on SDS polyacrylamide upon dissolution of the nanoparticles with 1% NaCl. The protein was observed at its molecular weight of 83KDa, and no protein degradation was observed, as seen in Figure 1F.

### Immune Response Generated in Mice by TMC-PA Alone and Upon Adjuvanation with CpG and Poly I:C

The immune response was elucidated in terms of the total IgG titer raised against the antigen. Since the mice were immunized *via* three routes: subcutaneous, IM, and IP; therefore, the comparison between different groups was also carried out within one frame of the route at a time.

### Total IgG Titer

**Figure 2:**
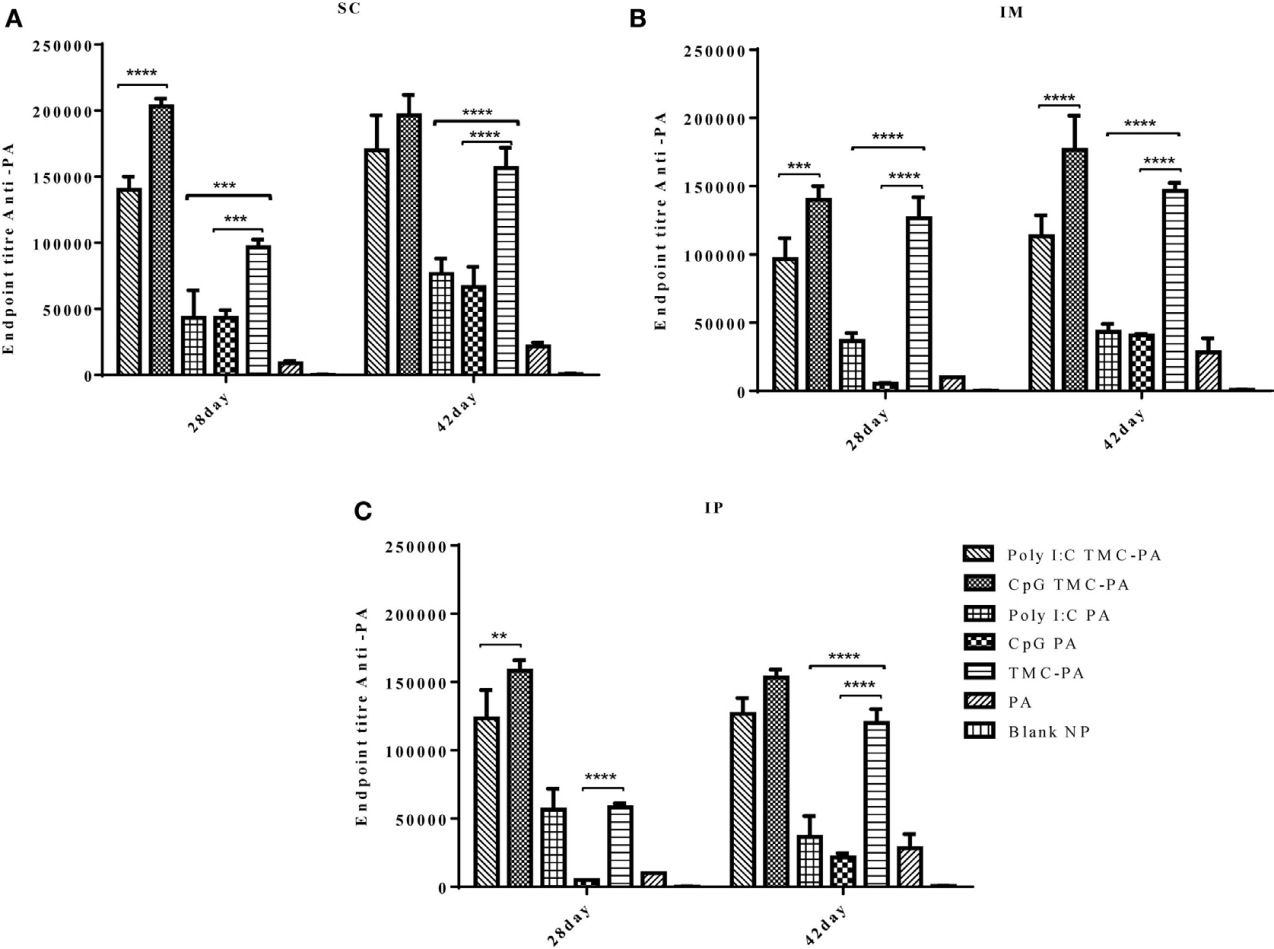
Serum antiprotective antigen (PA) total IgG endpoint titer. Female Balb/c mice (6–8 weeks of age; *n* = 21) were immunized with 1× PBS, PA, trimethyl-chitosan (TMC) NP-PA, Poly IC PA, Poly I:C TMC NP-PA, CpG PA, and CpG TMC NP-PA *via* three routes: subcutaneous (SC), intramuscular (IM), and intraperitoneal (IP) with a prime dose and two booster doses with an interval of 7 and 15 days, respectively. The mice were bled on 28th and 42nd day and sera samples were collected from all mice. Serial dilutions of sera from each group were analyzed for anti-PA total IgG titer for **(A)** SC route, **(B)** IM route, and **(C)** IP route. Statistically significant differences between the groups are highlighted (* for *P*-value <0.05, ** for *P*-values <0.01, *** for *P*-values <0.001 and **** for *P*-values <0.0001).

Figure 2 illustrates the highly elevated total IgG titer in almost all the vaccinated mice groups when compared to only PA vaccinated control mice. In all three routes, the descending order for the titers was CpG TMC-PA > Poly I:C TMC-PA > TMC-PA > Poly I:C PA > CpG PA. It could be seen that CpG and Poly I:C adjuvanation increased the total IgG titer from TMC-PA group significantly in almost all the three routes. In the subcutaneous route, the total IgG titer of the CpG TMC-PA group was significantly (*P* < 0.0001) higher than Poly I:C TMC-PA group on the 28th day (1.45-fold higher), whereas on 42nd day, the two titers were not significantly different. However in IM route, on both 28th and the 42nd day, the total IgG titer of CpG TMC-PA group is significantly (*P* < 0.01 and *P* < 0.0001, respectively) higher than Poly I:C TMC-PA group (1.44-fold on the 28th day and 1.5-fold on the 42nd day). Also, the TMC-PA, CpG TMC-PA, and Poly I:C TMC-PA groups showed significantly higher titer than the only PA group. These results elucidate the potential of TMC in mediating a very good humoral response in comparison to the only protein group and upon adjuvanation with CpG or Poly I:C, it increased in a synergistic manner. In all the three routes, the TMC-PA group showed significantly higher titer than CpG PA group and Poly I:C PA group (*P* < 0.0001). This shows that the TMC acts as a strong and better adjuvant in the form of nanoparticles with the PA protein and CpG as well Poly I:C adjuvanation improves the immune response mounted by TMC-PA alone.

### IgG Isotypes

**Figure 3:**
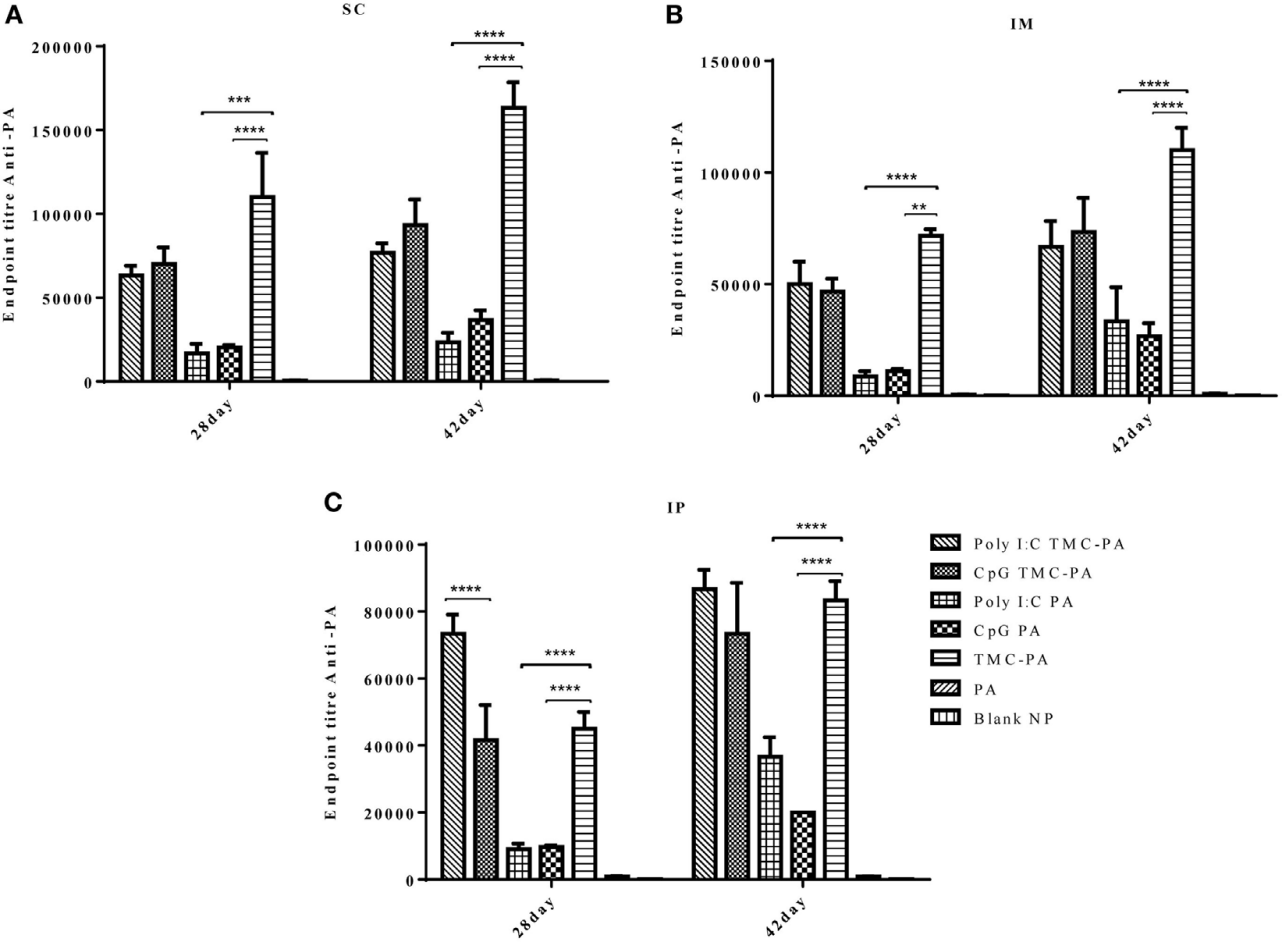
Serum antiprotective antigen (PA) IgG1 endpoint titer. Female Balb/c mice (6–8 weeks of age; *n* = 21) were immunized with 1× PBS, PA, TMC NP-PA, Poly IC PA, Poly I:C TMC NP-PA, CpG PA, and CpG TMC NP-PA *via* three routes: subcutaneous (SC), intramuscular (IM), and intraperitoneal (IP) with a prime dose and two booster doses with an interval of 7 and 15 days, respectively. The mice were bled on 28th and 42nd day and sera samples were collected from all mice. Serial dilutions of sera from each group were analyzed for anti-PA IgG1 titer for **(A)** SC route, **(B)** IM route, and **(C)** IP route. Statistically significant differences between the groups are highlighted (* for *P*-value <0.05, ** for *P*-values <0.01, *** for *P*-values <0.001, and **** for *P*-values <0.0001).

The levels of IgG1 (Figure 3) were higher in all TMC-PA containing groups in comparison to other groups, and this pattern was similar for all the three routes. On 28th and 42nd day, the TMC-PA group showed the highest titer in SC and IM route. However, for IP route, Poly I:C TMC-PA group showed the highest titer on both 28th and 42nd day followed by the TMC-PA group. The Poly I:C PA and CpG PA groups showed substantially lower levels of IgG1 in comparison to the other nanoformulation vaccinated groups at both the time points and all the routes (*P* < 0.0001).

**Figure 4:**
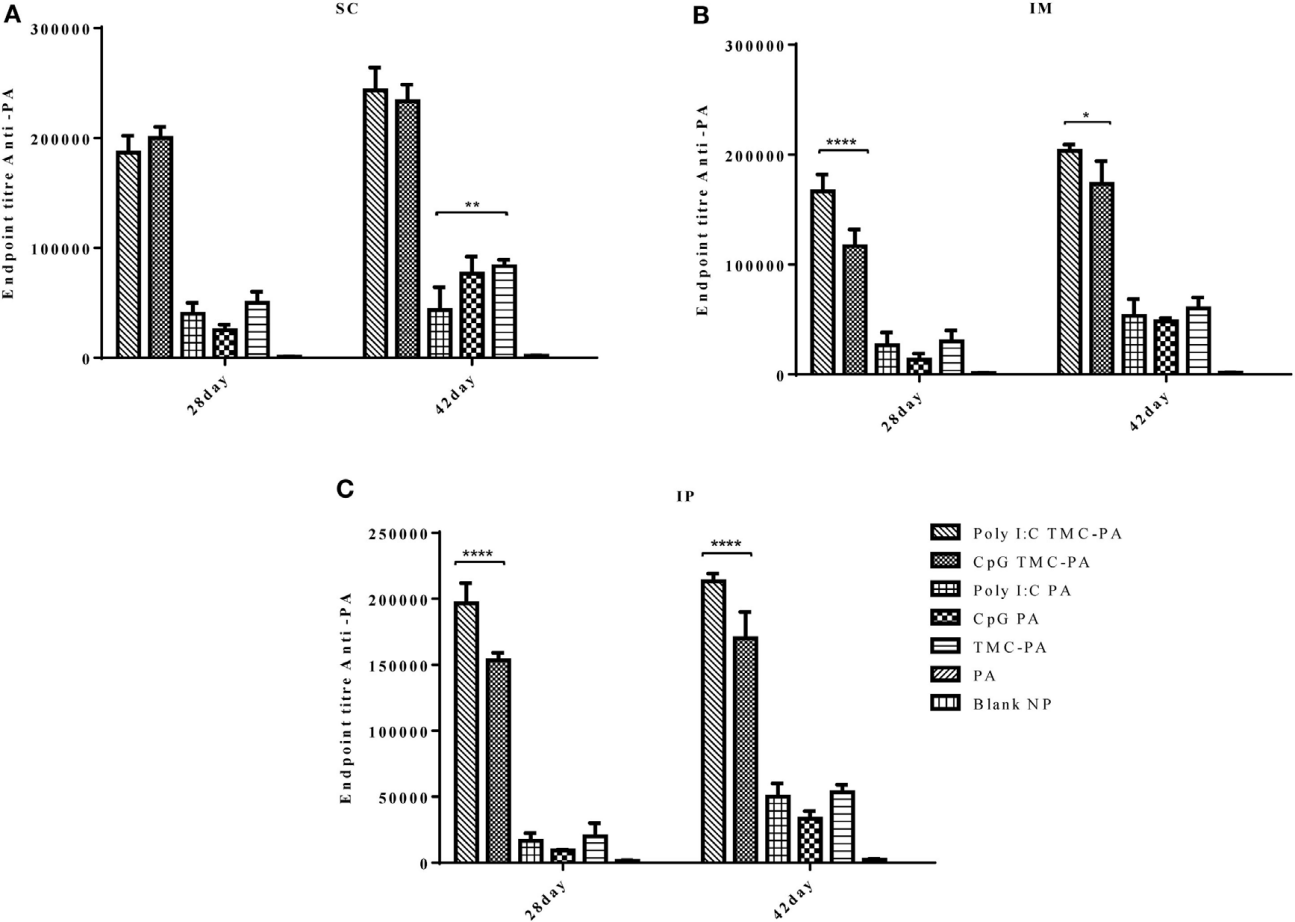
Serum antiprotective antigen (PA) IgG2a endpoint titer. Female Balb/c mice (6–8 weeks of age; *n* = 21) were immunized with 1× PBS, PA, trimethyl-chitosan (TMC) NP-PA, Poly IC PA, Poly I:C TMC NP-PA, CpG PA, and CpG TMC NP-PA *via* three routes: subcutaneous (SC), intramuscular (IM), and intraperitoneal (IP) with a prime dose and two booster doses with an interval of 7 and 15 days, respectively. The mice were bled on 28th and 42nd day, and sera samples were collected from all mice. Serial dilutions of sera from each group were analyzed for anti-PA IgG2a titer for **(A)** SC route, **(B)** IM route, and **(C)** IP route. Statistically significant differences between the groups are highlighted (* for *P*-value <0.05, ** for *P*-values <0.01, *** for *P*-values <0.001, and **** for *P*-values <0.0001).

In case of IgG2a subtype (Figure 4), among all routes, on the 28th and 42nd day, the Poly I:C TMC-PA and CpG TMC-PA groups showed the highest titers. The TMC-PA, CpG PA, and Poly I:C PA groups showed significantly lower titers when compared to the CpG and Poly I:C adjuvated TMC-PA groups, through all the routes, and at 42nd day, there was no significant difference in their titers as well, except in SC route. IgG isotypes have been of importance as to determine the Th biasness of the immune response. TMC has been reported to have a Th2-directing effect on the immune response, whereas CpG and Poly I:C have been reported to have a Th1 biasing effect on the immune response.

### Cytokine Release and Th1/Th2 Immune Response in Mice Groups

#### CpG TMC-PA Group

**Figure 5:**
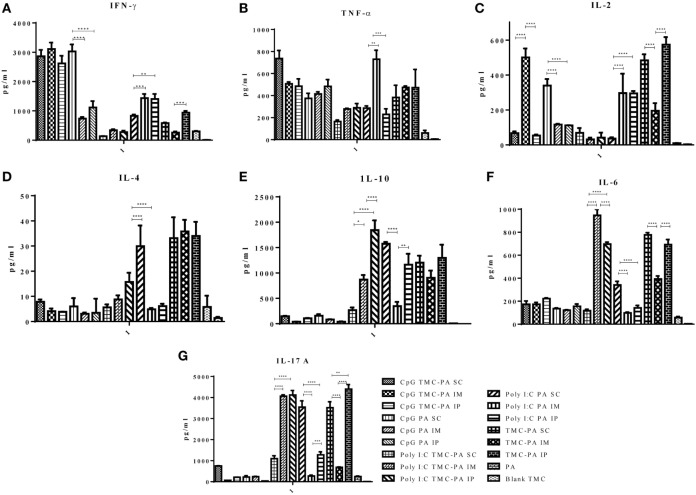
Cytokine levels. Female Balb/c mice (6–8 weeks of age; *n* = 21) were immunized with 1× PBS, PA, Trimethyl-chitosan (TMC) NP-protective antigen (PA), Poly IC PA, Poly I:C TMC NP-PA, CpG PA, and CpG TMC NP-PA *via* three routes: subcutaneous (SC), intramuscular (IM), and intraperitoneal (IP) with a prime dose and two booster doses with an interval of 7 and 15 days, respectively. 42 days after the primary immunization, mice (*n* = 3) from each group were sacrificed, and their spleen single cell suspension was used for **(A)** IFN-γ, **(B)** TNF-α, **(C)** IL-2, **(D)** IL-4, **(E)** IL-10, **(F)** IL-6, and **(G)** IL-17A cytokines analysis. Statistically significant differences between the groups are highlighted (* for *P*-value <0.05, ** for *P*-values <0.01, *** for *P*-values <0.001, and **** for *P*-values <0.0001).

Based on the cytokine levels and the levels of IgG isotypes (IgG1 and IgG2a), it was seen that CpG TMC-PA SC/IM/IP groups of mice developed a dominant Th1-biased immune response. By 42nd day, the ratio of IgG2a vs IgG1 was found to be 2.4 for SC route, 2.3 for IM route, and 2.3 for IP route (Figures 3 and 4). The IFN-γ levels for these groups for SC, IM, and IP routes were highest among all the groups with no significant difference between them (Figure 5A). IL-4 levels for this group were <10 pg/ml for all three routes with no significant difference among them (Figure 5D). Similarly, IL-10 levels were <150 pg/ml and IL-6 levels were ~200 pg/ml, with no significant difference among all three routes (Figures 5E,F). TNF-α level for the SC, IM, and IP routes were ~500 pg/ml, with no significant difference between different routes (Figure 5B). However, IL-2 levels for IM route were significantly higher (*P* < 0.0001) than SC and IP routes (Figure 5C).

#### CpG PA Group

This group also showed a Th1-skewed response as the ratio of IgG2a:IgG1 for SC, IM, and IP routes were 2.1, 1.8, and 1.7, respectively at 42nd day (Figures 3 and 4). The IFN-γ levels were significantly different between SC vs IM route (*P* < 0.0001) and SC vs IP route (*P* < 0.0001); however, no significant difference was observed between IM vs IP routes (Figure 5A). The Th2 cytokine IL-4 was observed to have very low levels (<10 pg/ml) supporting the Th1 biasness (Figure 5D). TNF-α levels for SC, IM, and IP routes were ~400 pg/ml (Figure 5B). The IL-2 levels of the SC route were significantly higher in comparison to IM (*P* < 0.0001) and IP (*P* < 0.0001) routes (Figure 5C). IL-10 and IL-6 levels for all three routes were <160 pg/ml, with no significant difference amongst the three routes (Figures 5E,F).

#### Poly I:C TMC-PA Group

At 42nd day, the ratios of IgG2a:IgG1 for SC, IM, and IP routes were 3.2, 3.0, and 2.5, respectively, suggesting a dominant Th1 response (Figures 3 and 4). In contrast, the IFN-γ and IL-4 levels do not support a strong Th-1 response (Figures 5A,D). Similarly, the levels of TNF-α and IL-2 were relatively low and exhibited no significant difference between the routes (Figure 5B). Both IL-10 and IL-6 levels were relatively higher with the IL-10 levels of IP route significantly higher than SC (*P* < 0.0001) and IM route (*P* < 0.001) (Figures 5E,F). The IL-6 levels for SC, IM and IP routes also revealed a highly significant inter-route difference (*P* < 0.0001) (Figure 5F).

#### Poly I:C PA Group

At 42nd day, the ratios of IgG2a:IgG1 for SC, IM, and IP routes were 1.9, 1.6, and 1.4, respectively, suggesting a moderate Th1 response (Figures 3 and 4). Similarly, the IFN-γ levels were relatively high showing a significant difference between SC vs IM route (*P* < 0.001) and SC vs IP (*P* < 0.01) routes whereas the IL-4 levels for all routes were >20 pg/ml, also suggesting a moderate Th-1 response (Figures 5A,D). The levels of TNF-α for the IM route were significantly higher than SC (*P* < 0.001) and IP (*P* < 0.0001) routes (Figure 5B). The IL-2 levels of IM and IP routes were found to be significantly higher (*P* < 0.0001) than SC route (Figure 5C). In contrast to the moderate Th-1 response demonstrated by above parameters, IL-10 values of SC and IP routes were higher, whereas IM route response was moderate (Figure 5E). The IL-6 levels for all three routes were observed to be <350 pg/ml (Figure 5F).

#### TMC-PA Group

At 42nd day, the ratios of IgG1:IgG2a for SC, IM, and IP routes were 2.9, 3.5, and 1.6, respectively, suggesting a dominant Th2 response (Figures 3 and 4). However, IFN-γ levels and IL-4 levels suggest a Th1 biased immune response (Figures 5A,D). As the values of IL-10 and IL-6 fall in the range of 500–1,200 pg/ml, it suggests a Th2 response, which is also supported by low levels (<500 pg/ml) of TNF-α and IL-2 (Figures 5B–F).

#### IL-17 A Cytokine

Figure 5G illustrates that the highest levels of IL-17A were observed in the TMC-PA IP group, followed by Poly:IC TMC PA IP group and Poly:IC TMC PA IM being ~4,000 pg/ml. The Poly:IC PA SC and TMC-PA SC groups produced similar levels of this cytokine. Poly:IC PA IP and Poly:IC TMC PA SC groups secreted ~1,000 pg/ml of IL-17. All the remaining groups secreted relatively lower amount of cytokine.

#### Survival Curve

**Figure 6:**
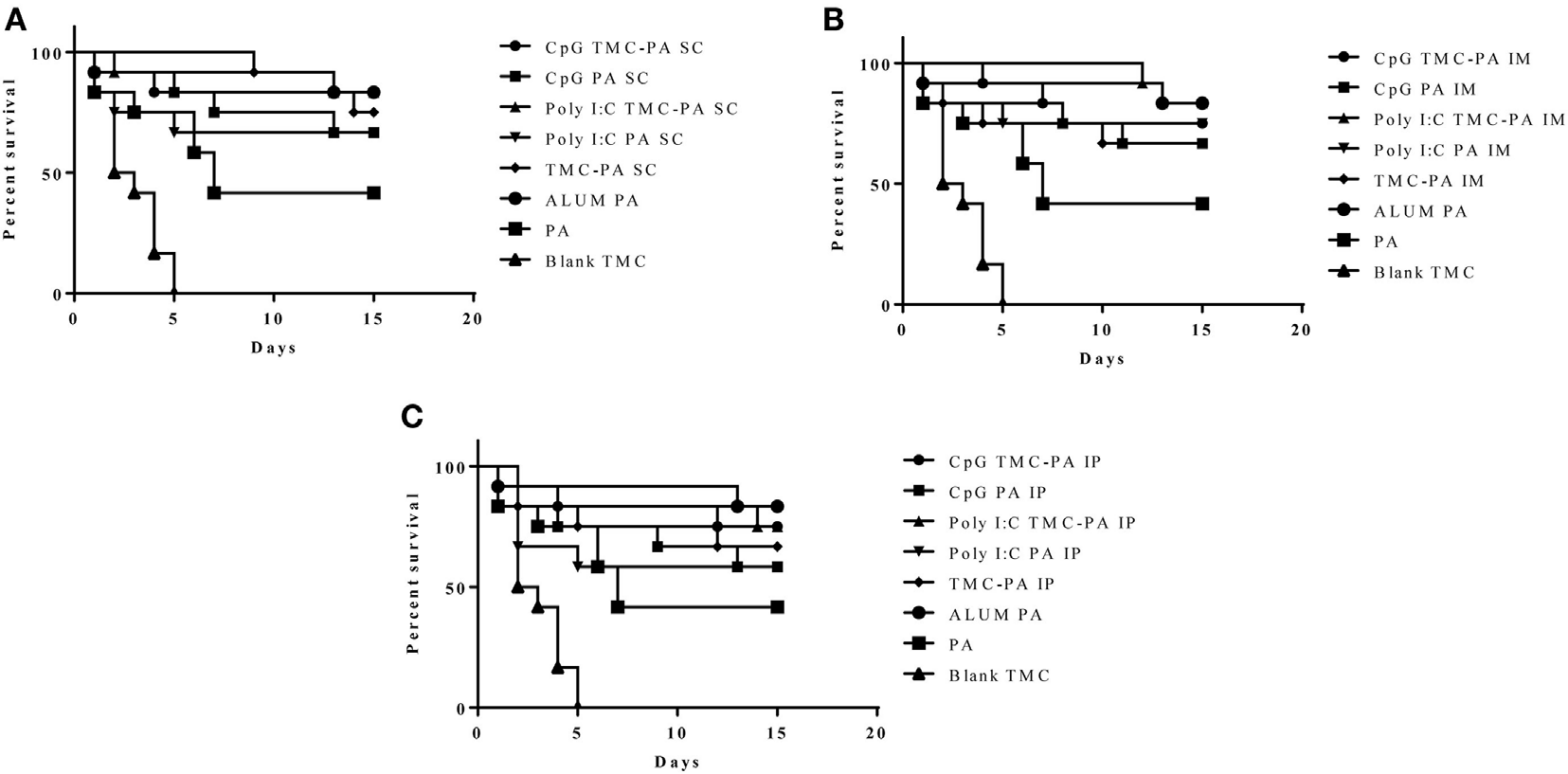
Survival curve. Female Balb/c mice (6–8 weeks) were immunized with 1X PBS, Protective Antigen (PA), Trimethyl-chitosan (TMC) NP-PA, Poly IC PA, Poly I:C TMC NP-PA, CpG PA and CpG TMC NP-PA *via* three routes: **(A)** subcutaneous (SC), **(B)** intramuscular (IM), and **(C)** intraperitoneal (IP) with a prime dose and two booster doses with an interval of 7 and 15 days, respectively. At 43rd day, mice in each group (*n* = 12) were challenged with 0.5 × 10^3^ spores of Ames strain of *Bacillus anthracis*. Mice were monitored for 15 days for death events in each group. Survival curve was plotted to compare the efficiency of protection in vaccinated mice groups over control placebo nanoparticle immunized mice group. As a control, 20 µg PA adsorbed on Alum (group named as ALUMN PA) was subcutaneously injected into a separate mice group (*n* = 12). The survival curve for anthrax spore challenge experiments was evaluated using Kaplan–Meier survival estimates (GraphPad Prism, La Jolla, CA, USA).

After 6 weeks of immunization, all the mice groups were challenged with 0.5 × 10^3^ spores of *B. anthracis* Ames strain. The mice were monitored for 2 weeks to observe the protective efficacy of different formulations as shown in Figure 6. The mice vaccinated with Alum and PA were taken as a positive control and exhibited a survival of 83.3%, which was the maximum of all the groups examined. The CpG TMC-PA SC, Poly I:C TMC-PA SC, and Poly I:C TMC-PA IM groups also showed a survival rate of 83.3%, equivalent to the positive control used. Except for the blank nanoparticles and only PA-treated groups, all other mice groups showed a survival rate of more than 50%. The TMC-PA SC group showed a survival of 75% and the TMC-PA IM and TMC-PA IP groups showed a survival of 66.6%. Our study demonstrates that the SC route of immunization showed a better survival rate in comparison to the other two routes.

## Discussion

The need to expand the arsenal of adjuvants and development of new subunit vaccine against anthrax with fewer side effects has set out an exploration of the natural, biodegradable polymers with enhanced bioavailability. This study investigated a novel combination of TMC nanoparticles with PA as a potential vaccine candidate against anthrax. To the best of author’s knowledge, this novel approach has not been reported to-date.

The preparation of TMC and its nanoparticles, its subsequent characterization of physicochemical properties, and *in vitro* release kinetics was studied before performing the murine studies. The synthesis of TMC polymer followed an intermediate step of formation of dimethyl chitosan. This method was adapted to avoid the O-methylation of the chain and chain scission which occurs commonly due to prolonged harsh conditions during the preparation of TMC. A DQ of >30% and lower extent of O-methylation enhances the water solubility of TMC. This property of TMC was leveraged for the preparation of PA-loaded TMC nanoparticles at pH 7 in HEPES buffer (17, 44). The TPP-based ionic gelation method follows the principle of complexation of oppositely charged molecules (protonated amine of TMC and TPP anion), resulting in the formation of nanoparticles (19, 47, 48). Unlike the widely used methods utilizing organic solvents and high temperature, the ionic gelation method is very mild, and thus we did not observe any degradation products in the SDS PAGE analysis (49–51).

The average size of PA-loaded TMC nanoparticles (254 nm) was found to be suitable for an efficient uptake by APCs. As the TMC nanoparticles were positively charged at physiological pH, an electrostatic interaction with the negatively charged cell membrane is highly likely. This is in alignment with a previously reported study where insulin, ovalbumin (OVA), and tetanus toxoid-based chitosan nanoparticles were shown to have a similar particle size, charge distribution (47, 52–54). In terms of the release kinetics of protein, the observed pattern of an initial burst release followed by a controlled release is also supported by other studies (52, 53). Thus, the *in vitro* antigen release study confirms the long-term antigen-releasing capacity of PA-TMCs without any protein degradation post-encapsulation.

After successful preparation and characterization of TMC nanoparticles, mice studies were conducted. Although the nasal route has been extensively explored for TMC nanoparticle-based immunization (19, 23, 47, 52, 55), we did not achieve a satisfactory immune response modulation. This could potentially be attributed to the large size of TMC-PA nanoparticles and subsequently its poor diffusion across the epithelial layer covering nasal-associated lymphoid tissue. In a similar instance, Boonyo et al. and Hagenaars et al. reported a subdued intranasal immune response for an OVA and HI-loaded TMC nanoparticle system (52, 55). Thus, the SC, IM, and IP routes were subsequently pursued for immunization.

The results of our study, as evident from Figures 2–6, demonstrated an effective immune response for TMC nanoparticles by all three routes (SC, IM, and IP). The striking results were that the TMC-PA groups *via* all routes were able to produce a sufficient amount of anti-PA titer as well as provided nearly 60–70% protection in anthrax challenged mice. More interestingly, the adjuvants CpG and Poly I:C behaved synergistically with TMC-PA in terms of generating an elevated anti-PA antibody titer and survival efficacy of nearly 70–80%. However, SC route was found be more effective in generating the highest IgG titer. Such response could be attributed to longer persistence, slower clearance, and prolonged antigen presentation after SC administration (56). Consequently, we could relate to the large number of vaccines, which are currently being administered *via* SC route like mumps, rubella, yellow fever, Japanese encephalitis, measles, Haemophilus influenza type b, inactivated polio vaccine, and *S. pneumonia* (25, 56).

In line with the previously reported studies, the TMC-PA group generated a Th2-biased immune response (25, 34). On the other hand, Poly I:C and CpG are reportedly Th1 biasing adjuvants (57–60). CpG alone has been reported to establish a Th1-biased immune response with several antigens (61–63). In this study, Poly I:C administration modulated the immune response toward Th1 with PA and TMC-PA nanoparticles. Supporting the earlier evidence, the CpG PA groups generated a Th1 response *via* all the three routes. Despite the fact that the PA is a Th2 biased antigen and TMC is also a Th2 biased adjuvant, the CpG adjuvated TMC-PA-treated groups also generated a dominant Th1 response *via* all the three routes. This observation is supported by Slütter and Jiskoot (34), which reported that the TMC-CpG nanoparticles encapsulating OVA as an antigen generated a 10-fold higher Ig2a titer than the TMC-TPP nanoparticles encapsulating OVA, thus provoked a strong Th1 response. The same report also revealed that the antigen-stimulated splenocytes secreted elevated levels of IFN-γ. This was also observed in our study that the CpG PA and CpG adjuvated TMC-PA groups showed highest levels of IFN-γ. In another study by Wang et al., virus-like particles encapsulating CpG-gold nanoparticle conjugates elicited a strong IFN-γ secretion (64). Therefore, we conclude that CpG enhances the production of IFN-γ, upon adjuvanation with an antigen. However, the levels of IL-4 produced by the splenocytes were low for all the groups, but the TMC-PA group relatively secreted the highest IL-4 irrespective of the route of immunization. Although, the CD4+ and CD8+ T-cell count gives a clear indication about the Th1 or Th2 response, but in this study, we did not analyze these parameters. However, the cytokines levels do indicate the direction of biasness of the immune response.

IL-17 T-helper cells are well known to serve as a bridge between innate and acquired immunity. Upon sensing the cytokines in the mucosal sites, the memory effector subset of Th17 cells can transform into Th1 or Th2 phenotype. IL-17A is the hallmark cytokine of the proinflammatory Th cell subset of Th17. As evident from Figure 5, Poly I:C PA and Poly I:C TMC-PA groups generated high levels of IL-17A. The TMC-PA group also secreted high levels of IL-17A, except the IM route. In a study by Holm et al., Poly I:C was reported to stimulate the production of IL-17A and IL-21 directly and consequently, drive the human naïve CD4+ T-cells differentiation, whereas CpG did not affect IL-17A on either mRNA or protein level (65). Similarly, Vultaggio et al. reported a positive correlation between Poly I:C and 1L-17A production (66). The chitin-based adjuvants have also been reported to be an immune response modulator (67) with a size-dependent response on macrophage IL-17A production (28). Thus, the growing scientific evidence indicates that TMC plays a role in stimulating IL-17A production. However, the mechanism of action remains unclear and need further exploration.

CpG and Poly I:C are PAMP-based adjuvants and act by initiating a cascade of innate immune system stimulation by interacting with TLR-3 and TLR-9, respectively. However, the immune modulation mechanism behind TMC nanoparticles remains elusive. We hypothesize that this immune-modulated response is due to the positively charged TMC nanoparticles, which might act as “danger signal” for dendritic cells.

In conclusion, the PA-loaded TMC nanoparticles as well as CpG and Poly I:C adjuvanted TMC-PA formulations elicited strong IgG antibody response *via* SC, IM, and IP routes in mice. However, the SC route showed the strongest response for IgG titer and survival efficacy. The TMC nanoparticle formulations were able to protect the mice against anthrax challenge and were comparable in protective efficacy to alum. In our opinion, TMC-based formulations possess a great potential as a vaccine candidate against anthrax, and further studies are needed to explore its adjuvant mechanisms.

## Ethics Statement

All mice experiments were performed in compliance with Institutional Animal Ethics Committee (Jawaharlal Nehru University) and Council for the Purpose of Control and Supervision of Experiments on Animals (CPCSEA, Ministry of Social Justice and Empowerment, Government of India). Mice were housed in the individually ventilated animal caging system.

## Author Contributions

Experiments conceptualized and designed by AM. Performed by AM, MG, and RM. Data analysis by AM. Wrote the article: AM, HG, and RB. Supervised by RB.

## Conflict of Interest Statement

The authors declare that the research was conducted in the absence of any commercial or financial relationships that could be construed as a potential conflict of interest.
