# Supplementary material for: Trimethyl Chitosan Nanoparticles Encapsulated Protective Antigen Protects the Mice Against Anthrax
Source: Front Immunol. 2018 Mar 20;9:562. doi: 10.3389/fimmu.2018.00562 (PMC5870345; doi:10.3389/fimmu.2018.00562)
Supplement: Supplementary file 1 [file data_sheet_1.DOCX]

**Trimethyl Chitosan Nanoparticles Encapsulated Protective Antigen**

**Protects the Mice Against Anthrax**

**Supplementary Material**

**Table S1**: Study design detailing injection volumes for each route of administration and anatomical site.

| **Route of administration** | **Anatomical site** | **Injection volume (μl)** |
| --- | --- | --- |
| Intramuscular (IM) | Quadriceps muscle | 100 |
| Subcutaneous (SC) | Neck/ Mid-belly | 100 |
| Intraperitoneal (IP) | Peritoneal cavity | 100 |


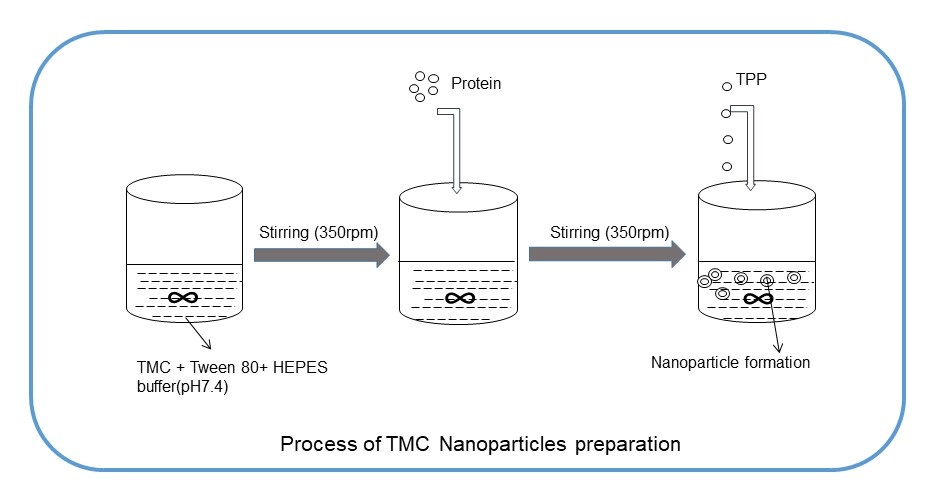


**Figure S1**: Schematic representation of the preparation of TMC nanoparticles encapsulating Protective antigen by TPP based ionic gelation method.


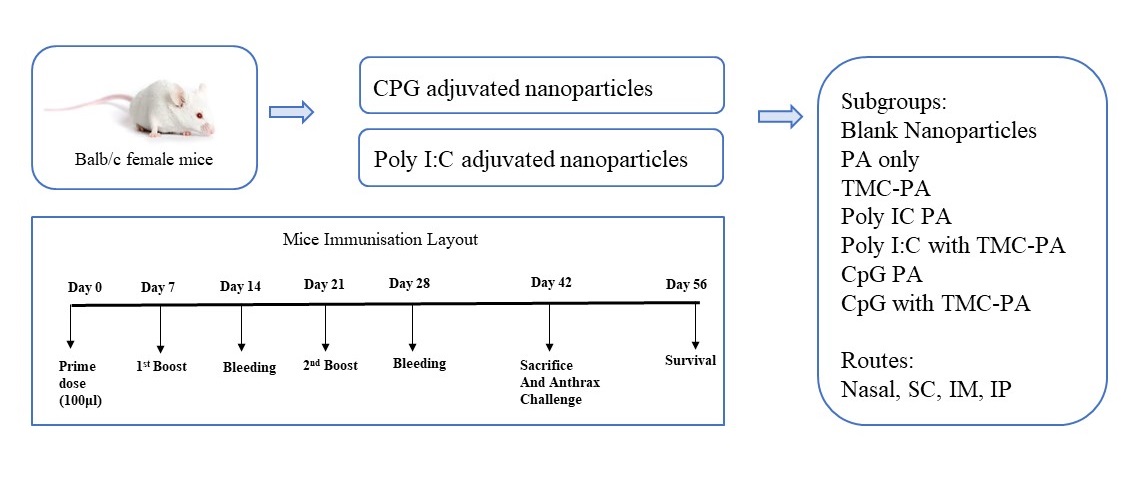


**Figure S2**: Mice immunization schedule. Female Balb/c mice (6-8 weeks of age) were immunized with the placebo nanoparticles, PA-TMC-NPs, PA-TMC-NPs in combination with CpG ODN as an adjuvant, and PA-TMC-NPs in combination with Poly I:C as an adjuvant. All these formulations named as 1X PBS, Only PA, TMC NP-PA, Poly IC PA, Poly I:C TMC NP-PA, CpG PA, and CpG TMC NP-PA were administered via three routes: Subcutaneous (SC), Intramuscular (IM) and Intraperitoneal (IP) in the mice. The amount of PA administered was 20µg whether encapsulated in nanoparticles or administered with CpG ODN and Poly I:C. Mice were immunized with a prime dose and two booster doses with an interval of 7 and 15 days respectively. The mice were bled on 28^th^ and 42^nd^ day and sera samples were collected from all mice. 42 days after the primary immunization, 3 mice from each group were sacrificed and their spleen single cell suspension was used for cytokine analysis. At 43^rd^ day all remaining mice in each group were challenged with 0.5x10^3^ spores of Ames strain of *Bacillus anthracis*. Mice were monitored for 15 days for death events in each group.


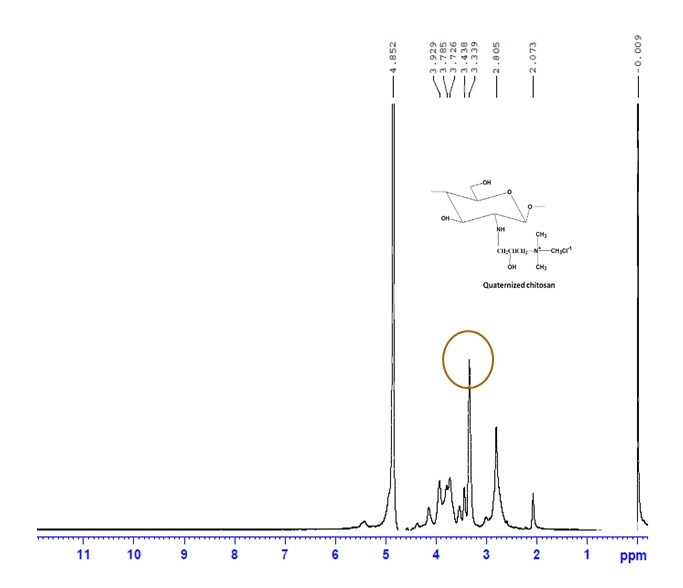


**Figure S3**: ^1^H-NMR spectrum of chitosan quaternary salt (TMC) dissolved in D_2_O.
